# Contributions of Microscopy to the Morphological Characterization of the Male Genitalia of *Toxomerus politus* (Diptera, Syrphidae)

**DOI:** 10.1002/ece3.70911

**Published:** 2025-02-02

**Authors:** Ingrid G. M. Silva, Alexandre A. F. Souza, Ariane P. Silveira, Karine B. Barros‐Cordeiro, Welinton R. Lopes, Sonia N. Báo

**Affiliations:** ^1^ Laboratório de Microscopia e Microanálise, Instituto de Ciências Biológicas Universidade de Brasília Brasília Brazil; ^2^ Instituto de Biologia Universidade Federal de Uberlândia, Campus Umuarama Uberlândia Minas Gerais Brazil; ^3^ Laboratório de Insetos Necrófagos, Instituto de Ciências Biológicas Universidade Federal de Goiás Goiania Brazil

**Keywords:** confocal laser scanning microscopy, critical point, SEM, TEM, *Toxomerus politus*, ultrastructure

## Abstract

The study of insects has historically been linked to the development of microscopy. As techniques have improved, research into insect morphology can increasingly contribute to a better understanding of the structures and functions of the morphological characters of insects' three tagmata. We propose the use of methodologies applied in microscopy as a way of improving and facilitating the study of the morphological characters of fly genitalia. In addition, flies preserved in 70% alcohol were used to verify the feasibility of distinguishing ultrastructural characters in the spermatozoa, when not previously fixed for the preservation of morphological structures. We have shown that it is viable to enhance preservation of specimens using a scanning electron microscopy technique. In addition, the observation of genitalia using this technique, together with confocal laser scanning microscopy, enables better visualization, description, and understanding of the morphological characteristics of fly genitalia. We also noted, in analysis using transmission electron microscopy, that it is possible to recover and describe some morphological features of the ultrastructure of *Toxomerus politus* (Say, 1823) sperm, obtained from flies stored in 70% alcohol for a long period of time. Comparison between the methods used to investigate the structures of *T. politus* , observed after preserving the specimen, already demonstrates the importance of using appropriate methodology as a starting point for reliable results. The methodologies and techniques adopted in this work have the potential to be extrapolated to research into other species of flies and other insects in a broad context.

## Graphical abstract

The use of microscopy as a morphological analysis tool in *Toxomerus politus* (Diptera, Syrphidae). A scientific‐artistic characterization of the male genitalia of Syrphidae.

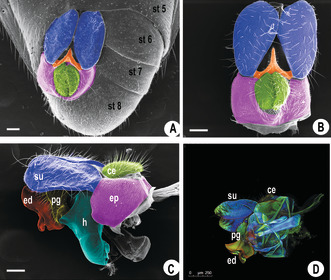

## 1 Introduction

The identification and classification of insects depends on the evaluation of tiny structures, which are often impossible to differentiate without technical equipment, such as stereomicroscopes and light microscopes.

The use of microscopy has helped advance entomological discoveries over the years. In 1669, the naturalist, anatomist, and microscopist Jan Swammerdam (1637–1680) demonstrated how microscopy could be used as a powerful tool in the study of insects. He published his most famous book, “Historia Insectorum Generalis” (The Natural History of Insects), in which he used progressive techniques for dissecting and comparing structures to discuss the development and metamorphosis of insects (Friedrich et al. 2014). As technologies improved, including the use of stereomicroscopes, entomologists were able to identify and differentiate morphological and structural characters, which are essential for taxonomy. Antennae, wings, and legs, for example, saw their morphologies and functions properly described, which enabled a better understanding of abilities such as communication and flight (Callahan 1975; Farisenkov et al. 2022).

With the emergence of electron microscopy, the power of resolution was increased, enabling the visualization of structures on micro and nanometric scales. From the 1970s onwards, studies involving entomology and the use of scanning electron microscopy (SEM) began to be developed (Conford, Rowley, and Klun 1973; Wootton 1992). Structures and external morphological characters previously described, with less detail, or unknown, became part of morphological descriptions and taxonomic studies (Wootton 1992; Dey, Hooroo, and Wankhar 1995).

Transmission electron microscopy (TEM), in turn, has helped to understand the role of the structure and function of cells and tissues, which is essential, for example, in the ultrastructural evaluation of gametes (Stirling and Woods 2002; Ravi, Leung, and Zeev‐Ben‐Mordehai 2020). The study of the morphology and ultrastructure of spermatozoa has proven to be relevant since the diversity of sperm morphology can provide data on the evolutionary path (Higginson 2011; Rezende et al. 2024) and potential mechanisms related to variability in the fertilization and reproduction of species (Malawey et al. 2019). When data on spermatozoa morphological characters are analyzed alongside other information, such as molecular data and external morphology, the results become consistent and reliable, enhancing our understanding or phylogenetic relationships (Gottardo et al. 2016; Tavares‐Bastos et al. 2024).

Confocal laser scanning microscopy (CLSM), on the other hand, through natural or artificial fluorescence, has allowed even more accurate differentiation of insect anatomy, providing information about diversity, adaptation, and evolutionary relationships (Lee, Brown, and Monroe 2009), making it possible to visualize features from characteristic structural profiles (Zhang et al. 2013).

Regardless of the aforementioned possibilities and potentialities, there are still challenges, since insects such as flies and mosquitoes often have fragile bodies and structures (Sarwar 2020). The methodology commonly used to preserve specimens, where the insects are dried and mounted at room temperature, sometimes fails to maintain the integrity of their original characteristics and the conservation of morphological structures (e.g., loss of color, collapse of the integument) (author's observation). Another issue is the lack of reliable representation of morphological structures, which are often represented by schematically inaccurate and confusing drawings, especially regarding the genitalia of males (e.g., Metz and Thompson 2001; Borges and Couri 2009).

In insects, male genitalia is an essential morphological character for taxonomists, fundamental for differentiating closely related species (Eberhard 2019), as it shows great diversity and variability when compared to other insect body structures (Sinclair, Cumming, and Wood 1993; Sinclair, Cumming, and Brooks 2013). Likewise, spermatozoa and females can contribute to the understanding of insect phylogeny. After mating, sexually mature females retain these cells in specialized storage organs called spermathecae (Name, Pujol‐Luz, and Báo 2010).

One group that represents a taxonomical challenge and could benefit from more reliable approaches to the preservation and representation of its structures is the family Syrphidae (Diptera). These flies constitute a speciose group with at least 7000 described species (Catalogue of Life 2024) and are highly diverse in morphology, comprising both large‐bodied flies, and smaller, slender ones, such as those in the genus *Toxomerus* Macquart, 1855.

Moreover, syrphid flies can provide ecological services such as pollination when adults (Golding and Edmunds 2000; Feldman 2006; Jauker and Wolters 2008; Rader et al. 2009), and decomposition of plant matter when still immature. They can also be used as bioindicators (Gilbert 1993), and some species are of public health concern since they can cause Myiasis (Aguilera et al. 1999) and, when in degraded environments, act as mechanical vectors of pathogens (Francuski et al. 2011).

Despite the diversity and importance of Syrphidae, accurate species identification is challenging. It is dependent on chaetotaxy and the color patterns on the wings, abdomen, and thorax (Metz and Thompson 2001; Borges and Couri 2009), which are often damaged or lost with traditional methods of mounting and preservation. Additionally, the utilization of individual or combined microscopy techniques, while well established, is not commonly used for the processing of entomological samples, especially of syrphids (Harbach 1984; Metz and Thompson 2001; Mengual 2011), which are mostly described with illustrations and mounted with traditional methods.

Here we combine distinct microscopy techniques (SEM, TEM, and CSLM) to preserve and describe fragile structures. A species of the genus *Toxomerus* from the Syrphidae family was used as a model: it is a small, slender insect, with several specific and easily damaged morphological markers, such as abdomen coloration. Also, we present information on the structure and ultrastructure of the male genitalia of our model species.

## 2 Materials and Methods

### 2.1 Obtaining the Material

Flies of the species *T. politus* (Say, 1823), belonging to the Syrphidae family (Diptera), were chosen to demonstrate the applied techniques and their outcomes since they are flies with a fragile and delicate structure (as observed by the authors and Sarwar 2020).

Prior to this study, the specimens had been stored in 70% ethanol for approximately 5 years. They were collected in protected areas of Cerrado at the Emas National Park (Mineiros‐GO) and Silvânia National Forest (Silvânia—GO), deposited at ZUFG (Zoological Collection of the UFG); and Fazenda Água Limpa (Brasília‐DF), deposited at DZUB (Entomological Collection of the Department of Zoology of the University of Brasília). Species were identified using dichotomous keys (Metz and Thompson 2001; Marinoni, Morales, and Spaler 2007; Borges and Couri 2009).

#### 2.1.1 Dissection of Genitalia

The abdomens of the males (*n* = 10) were removed and placed in 10% KOH solution for 24 h at room temperature to whiten and eliminate soft tissue. The genitalia were then dissected and subsequently prepared for microscopy analysis.

The dissected and analyzed specimens were discarded and those that were not dissected were deposited in the DZUB collection.

### 2.2 Comparison of Drying Methods

To compare outcomes of distinct drying methods with regard to the preservation of diagnostic morphological characters, specimens were mounted using entomological pins and dried either at room temperature or using part of the SEM protocol, with acetone dehydration and critical point drying (Gordh and Hall 1979).

Once they were completely dry, the flies were photographed using a Canon EOS Rebel T7 camera with a Canon EF 100 mm macro lens. The images were post‐processed with Darktable software version 4.4.2. Focus stacking was done in Helicon Focus version 8.0.2.

### 2.3 Confocal Laser Scanning Microscopy (CLSM)

The male genitalia were mounted on glass slides with glycerol and a coverslip. The samples were analyzed using a Laser Scanning Confocal Microscope, model TCS SP5 (Leica, Germany), equipped with lasers with the following wavelengths: 408, 488, and 594 nm. The autofluorescence signal was captured in 3 PMT channels with the following windows: blue (410–470), green (500–545), and red (600–720). The structures were analyzed using the ×10 objective with a dry lens. Sequential images were obtained using the Z‐stack and subsequently visualized using 3D projection.

### 2.4 Scanning Electron Microscopy (SEM)

The male genitalia were fixed in Karnovsky solution (2% glutaraldehyde, 2% paraformaldehyde, 5 mM CaCl_2_, and 3% sucrose in 0.1 M sodium cacodylate buffer), pH 7.2, at 4°C for approximately 12 h. They were post‐fixed for 1 h in 1% osmium tetroxide, and then dehydrated in an increasing series of acetone (30%, 50%, 70%, 90%, and 100%) for 15 min each. The samples were dried in critical point equipment, model CPD 030 (Balzers, Liechtenstein) and metalized with gold with EM SCD 500 equipment (Leica, Austria). The analyses were carried out using a Jeol model JSM‐7001F (Jeol, Japan) scanning electron microscope.

### 2.5 Transmission Electron Microscopy (TEM)

Additionally, in an attempt to obtain better‐preserved spermatozoa, spermathecae were used; so, the male testicles (*n* = 10) and female spermathecae (*n* = 10) were removed from flies previously stored in 70% alcohol and fixed in Karnovsky solution, pH 7.2, at 4°C for approximately 12 h. They were post‐fixed for 1 h in 2% osmium tetroxide, 1.6% potassium ferricyanide in 0.2 M sodium cacodylate buffer, pH 7.2, then contrasted in a block in 0.5% aqueous uranyl acetate solution for 12 h, dehydrated in an acetone series (30%, 50%, 70%, 90%, and 100%), and embedded in Spurr resin.

The ultra‐thin sections were cut using an ultramicrotome, model EM UC7 (Leica, Austria) contrasted in uranyl acetate and lead citrate, and afterward examined and photographed using a Transmission Electron Microscope, model JEM‐1011 (Jeol, Japan) at 80 kV.

The electron microscopy protocols were adapted from De Souza (2015), Gracielle, Tidon, and Báo (2016), and Barros‐Cordeiro, Pujol‐Luz, and Báo (2021).

## 3 Results

**FIGURE 1:**
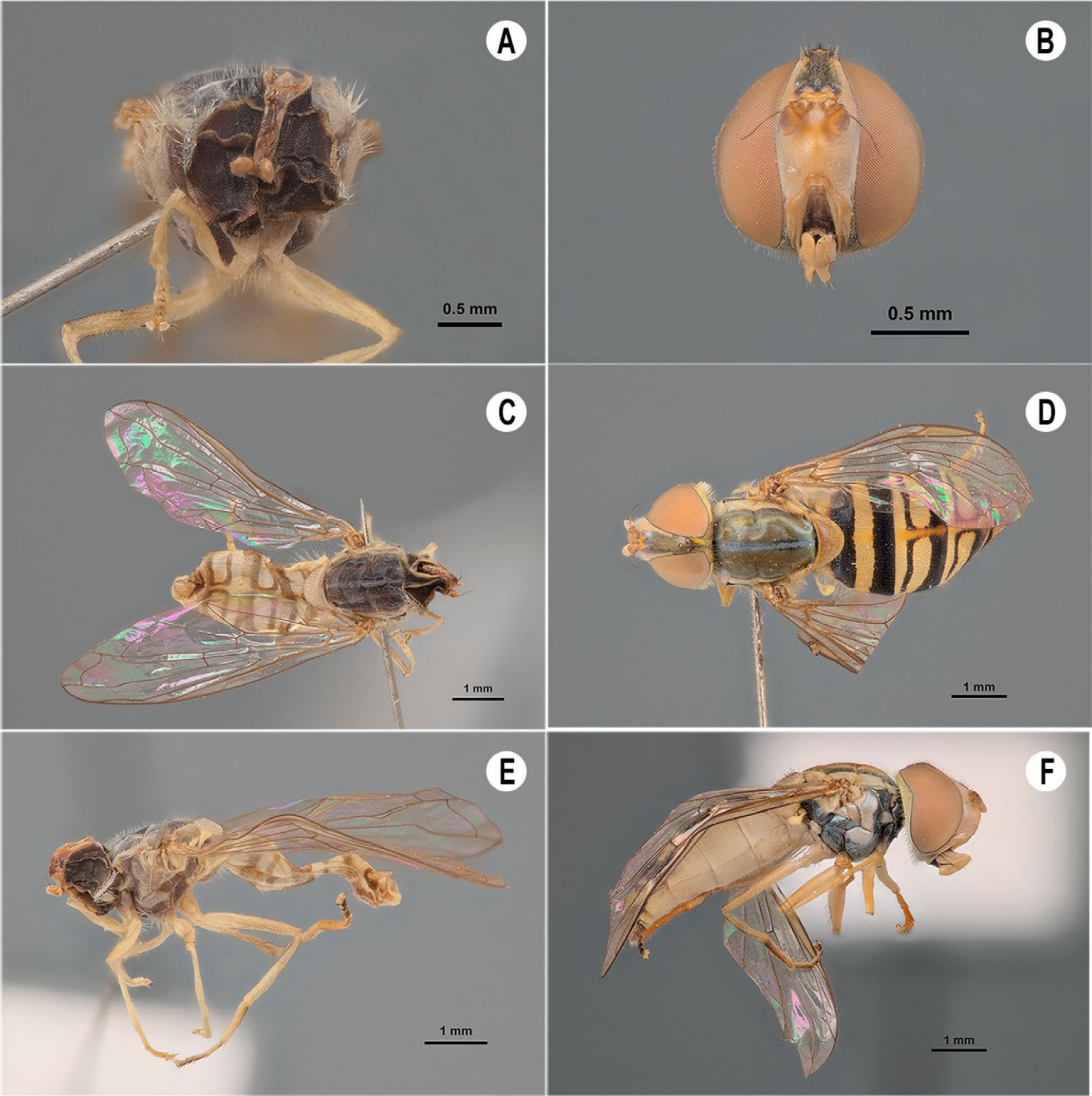
Comparison between two drying methods of the *Toxomerus politus* specimens: At room temperature (A, C, and E) and using the critical point drying protocol (B, D, and F).

Specimens submitted to the SEM protocol (dehydration and critical point drying) maintained a high degree of morphological integrity when compared to specimens dried at room temperature or using drying ovens. The most delicate structures, such as the head and eyes, as well as the abdomen, were not damaged when subjected to critical point drying; likewise, the specimen's original coloration was also maintained (Figure 1B,D,F). Conversely, flies dried at room temperature underwent discoloration and their integument collapsed, which led to a shriveled appearance and alteration in several morphological characters (Figure 1A,C,E).

### 3.1 Spermatozoa

**FIGURE 2:**
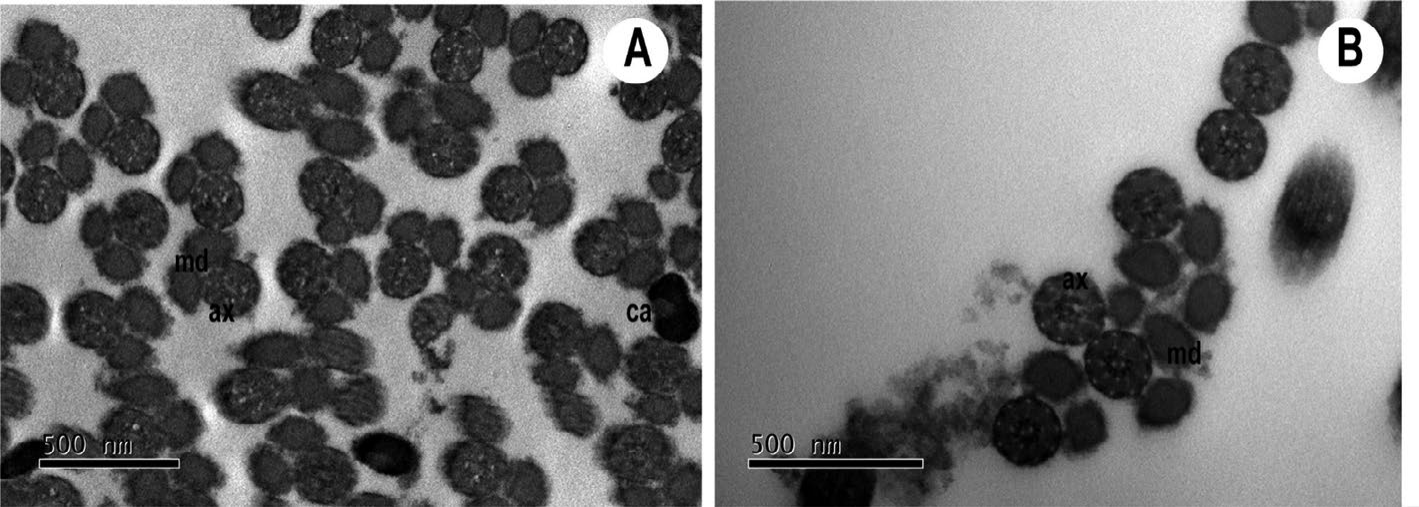
Transmission electron micrograph of *Toxomerus politus* sperm. Images (A) and (B) shows the morphology of structures such as the mitochondrial derivatives (md), the axoneme (ax), and the adjunct centrioles (ca).

Although the testicles and spermathecae had been taken from flies preserved in 70% alcohol, which could result in the deterioration of the tissues and cells, leading to some degree of degradation, it was still feasible to recover information about the spermatozoa morphology. The identification of some morphological features of the sperm ultrastructure, such as in the head (nucleus) and the tail (the centriole adjunct, the mitochondrial derivatives, and the axoneme) regions, was made using the TEM technique (Figure 2A,B). In addition, it was described how the transition from the head–tail region occurs with the insertion of the centriole adjunct.

### 3.2 Genitalia

**FIGURE 3:**
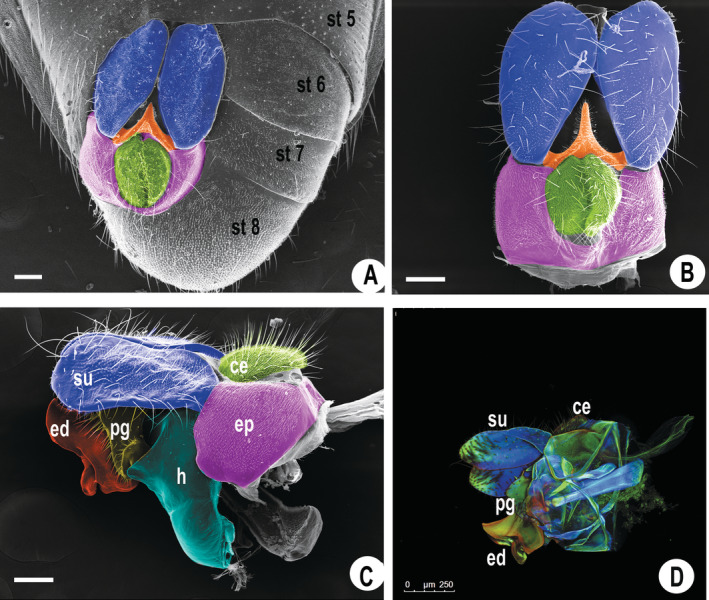
Scanning electron micrographs of the genitalia of *Toxomerus politus* and its structures. The sternites (st) are visible. The surstylus (su) region is highlighted in blue, the cercus (ce) in green, the epandrium (ep) region in purple, the hypandrium (h) in light blue, the postgonite (pg) region in yellow, the triangular process in orange and the edeagus (ed) in red (A–C; SEM). In D (CLSM) it is possible to see the insertion of the aedeagus behind the postgonite and epandrium. Scale bar at 100 μm (A–C), 250 μm (D).

The male genitalia were morphologically characterized using SEM, along with the adopted methodology. This allowed components such as the aedeagus, surstylus, cercus, and postgonite (Figure 3A–C) to be more prominently highlighted. It provided a more precise visualization of structural features, revealing ultrastructural information that is often unclear or inaccurately described with schematic drawings. Additionally, the presence of bristles and sensilla in different parts of the genitalia was identified.

Additionally, the CLSM technique enabled the characterization of genital structures through cuticular autofluorescence, highlighting color differences and enabling the visualization of specific features such as the insertion of the aedeagus and other previously inaccessible internal structures. In CLSM, three‐dimensional structures become translucent, revealing internal components hidden by external structures (such as the postgonite, epandrium, and hypandrium) that would otherwise require removal for observation (Figure 3D).

In 3D imaging using CLSM, this insertion can be clearly seen. The combination of both techniques (SEM and CLSM) enhances understanding of how internal structures interrelate and connect with each other.

## 4 Discussion

One of the major problems faced by researchers in the fields of entomology and taxonomy is the preservation of insects' structure. They can be fragile, and the standard protocol for drying specimens is carried out at room temperature or in incubators. In studies involving syrphids, damaged specimens with poorly preserved morphological structures are frequently observed (e.g., Reemer 2010; Carvalho‐Filho 2014; Miranda, Skevington, and Marshall 2020), but due to their significance in taxonomic studies, researchers make use of what can still be observed.

Critical point drying largely improved the overall specimen integrity, especially of delicate structures such as the eyes, head, and abdomen. The original coloration of the specimen was also maintained, which is a relevant factor, since color can be an important morphological character for the taxonomy of several insect taxa (e.g., Badejo et al. 2020; Cruz et al. 2021; Song et al. 2023).

While resource‐ and time‐consuming, the critical point drying method could be worthwhile to produce at least a few specimens that will be more easily identifiable in the future, especially when describing new species and assigning type specimens. The method allows the sample to remain intact, preserving the characteristics of the living insect. In this way, they can be deposited in entomological collections for future analysis.

Moreover, the techniques used in this work to characterize *T. politus* male genitalia allowed important structures to be visualized more clearly than when using images and schematic drawings obtained with a stereomicroscope, such as those seen in Thompson (1981).

These techniques provide a more reliable source of comparison between species and thus expand the morphological data currently available in the literature, regarding the genitalia of this species. Similar analyses carried out on other species can also be found. In the work conducted by Samerjai et al. (2021), two species of flies belonging to the Sarcophagidae family were identified, based on morphological and morphometric variations relating to the cercus and juxta (structural features of the male genitalia).

Buenaventura and Pape (2018) used morphological data from male genitalia from the subfamily Sarcophaginae to accomplish a phylogenetic analysis, in which SEM was also used to visualize the morphological characters for comparison. By SEM technique, analysis of the morphological details of the genitalia of *Parasacophaga dux* (Thomson, 1869) (Sarcophagidae) (Chaiwong et al. 2007) allowed the distinction between this and other species previously reported in the literature (Bänziger and Pape 2004).

In terms of the ultrastructure of spermatozoa, the literature traditionally reports the standard protocol of fixing the testicles immediately after dissection (Gracielle, Tidon, and Báo 2016; Malawey et al. 2019; Olazia et al. 2022), so that the structure of the spermatozoa is preserved. In studies that have analyzed the ultrastructure of insects, it was possible to characterize each of the morphological characters that compose the spermatozoa, thus providing a description and highlighting the morphological difference when comparing species (Name, Pujol‐Luz, and Báo 2010; Gracielle, Tidon, and Báo 2016; Mercati et al. 2023).

Kotzé et al. (2019) compared the ultrastructure of the sperm of *Hermetia illucens* (Linnaeus, 1758) (Stratiomyidae) with other species of Diptera, revealing distinct features in the head region, overlap zone (posterior part of the nucleus), and anterior flagellar region.

The morphological difference found in the ultrastructural analysis of sperm can also contribute to a better understanding of the diversity of reproductive strategies in insects (Pitnick, Hosken, and Birkhead 2008; Malawey et al. 2019).

The possibility of recognizing and characterizing some of the morphological features of the sperm ultrastructure, obtained from the testicles of flies stored in 70% alcohol, as conducted in this study, should be seen as an achievement and powerful resource since, in some cases, there may be a very small amount of material available for analysis and/or even rare specimens.

Additionally, the CLSM technique provided a complementary and improved visualization/ identification of morphological structures, which are naturally overlapping and challenging to differentiate when using light microscopy alone, offering a sophisticated elucidation of *T. politus* genital morphology. It included the visualization of multiple focal planes which, when combined, enable the three‐dimensional reconstruction of structures (Grzywacz et al. 2014).

Confocal laser scanning microscopy is still a tool rarely used in the morphological study of insects, but in the literature, we find studies demonstrating its importance in the morphological characterization of immature stages of Diptera (Grzywacz et al. 2014). For instance, Szpila et al. (2021) provided a morphological description of the cephalopharyngeal skeleton of first instar larvae. There are also other studies using CLSM for the three‐dimensional reconstruction of insect exoskeletons (Zill et al. 2000; Klaus, Kulasekera, and Schawaroch 2009).

## 5 Conclusion

Storing *T. politus* flies in 70% ethanol and drying them at the critical point enhanced the preservation of the specimens investigated, contributing to more reliable and detailed results regarding the structures and their morphologies evaluated.

The techniques used in this work presented specific and complementary results, identifying additional morphological characters, as shown through scanning electron and CLSM.

The techniques applied in this study can contribute to the fields of entomology and taxonomy. Although the use of SEM has already been reported in the literature as the best technique for the morphological study of insects, the literature does not provide comparative images showing these differences. Furthermore, this is the first work to provide a morphological description of the genitalia of *T. politus* via SEM and confocal microscopy and its sperm ultrastructure by TEM.

## Author Contributions

**Ingrid G. M. Silva:** conceptualization (lead), formal analysis (lead), investigation (lead), methodology (lead), writing – original draft (lead). **Alexandre A. F. Souza:** methodology (equal). **Ariane P. Silveira:** methodology (equal), writing – original draft (supporting). **Karine B. Barros‐Cordeiro:** conceptualization (equal), investigation (equal), methodology (equal), writing – review and editing (equal). **Welinton R. Lopes:** conceptualization (equal), investigation (equal). **Sonia N. Báo:** conceptualization (equal), investigation (equal), resources (lead), supervision (lead), writing – review and editing (lead).

## Conflicts of Interest

The authors declare no conflicts of interest.

## Data Availability

The authors confirm that the data supporting the conclusions of this study are available in the article.
